# Keeping it in the family: using protein family templates to rescue low confidence AlphaFold2 models

**DOI:** 10.1093/bioadv/vbae188

**Published:** 2024-11-25

**Authors:** Francesco Costa, Matthias Blum, Alex Bateman

**Affiliations:** European Molecular Biology Laboratory, European Bioinformatics Institute (EMBL-EBI), Hinxton CB10 1SD, United Kingdom; European Molecular Biology Laboratory, European Bioinformatics Institute (EMBL-EBI), Hinxton CB10 1SD, United Kingdom; European Molecular Biology Laboratory, European Bioinformatics Institute (EMBL-EBI), Hinxton CB10 1SD, United Kingdom

## Abstract

**Motivation:**

High confidence structure prediction models have become available for nearly all protein sequences. More than 200 million AlphaFold2 models are now publicly available. We observe that there can be significant variability in the prediction confidence as judged by plDDT scores across a protein family. We have explored whether the predictions with lower plDDT in a family can be improved by the use of higher plDDT templates from the family as template structures in AlphaFold2.

**Results:**

Our work shows that about one-third of the time structures with a low plDDT can be “rescued,” moved from low to reasonable confidence. We also find that surprisingly in many cases we get a higher plDDT model when we switch off the multiple sequence alignment (MSA) option in AlphaFold2 and solely rely on a high-quality template. However, we find the best overall strategy is to make predictions both with and without the MSA information and select the model with the highest average plDDT. We also find that using high plDDT models as templates can increase the speed of AlphaFold2 as implemented in ColabFold. Additionally, we try to demonstrate that as well as having increased overall plDDT, the models are likely to have higher quality structures as judged by two metrics.

**Availability and implementation:**

We have implemented our pipeline in NextFlow and it is available in GitHub: https://github.com/FranceCosta/AF2Fix.

## 1 Introduction

The introduction of AlphaFold2 (AF2) enabled the rapid and accurate modelling of protein structures (Jumper *et al.* 2021) and has made huge contributions to diverse areas of molecular biology. The AlphaFold Protein Structure Database (AFDB) (Varadi *et al.* 2022, 2024) is a huge collection of precalculated AF2-derived structure models, now containing over 200 million proteins. Also 600 million models calculated with ESMfold on metagenomic-derived protein sequences have been made available in the ESM Metagenomic Atlas (Lin *et al.* 2023). These collections mean that researchers can simply download high confidence models rather than predicting their own. A major strength of these structure predictions is that they come with reliable confidence metrics such as the predicted local difference distance test (plDDT) measure. The plDDT is a prediction from the Deep Learning model that predicts the lDDT score (Mariani *et al.* 2013), a superposition-free score that evaluates local distance differences of all atoms in a model. The lDDT score is particularly useful because unlike superposition-based methods it is generally unaffected by large scale domain orientation errors.

**Figure 1.**
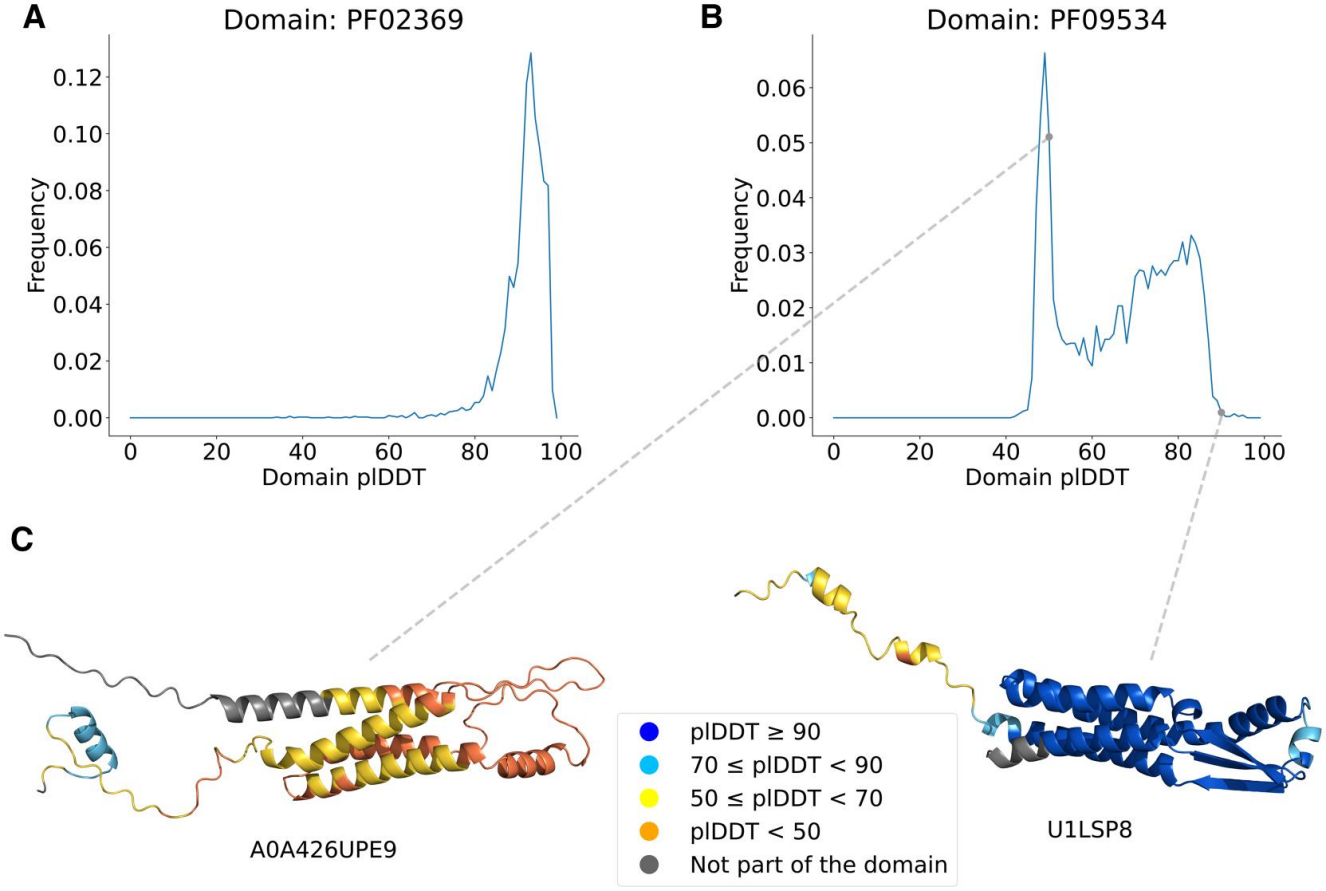
Distributions of the mean plDDT values taken from AFDB models for protein domains from Pfam families (A) Big_1 PF02369 and (B) family Trp_oprn_chp PF09534. Note that the mean plDDT values here are not for the whole protein length but rather the domain extent (grey regions are excluded). (C) Examples of AlphaFold2 models for family PF09534: low domain plDDT, UniProtKB: A0A426UPE9 and high domain plDDT, UniProtKB: U1LSP8.

Pfam is a collection of Hidden Markov models which are statistical models used to classify protein sequences into domains and families (Mistry *et al.* 2021). They are built based on multiple sequence alignments (MSAs) and manually curated. While browsing AFDB, we noticed that different proteins from the same protein family can have very different mean plDDT scores which prompted us to more systematically explore plDDT across protein families. Indeed, some protein families present significant variability among AlphaFold2 model plDDT. Figure 1 illustrates two example plDDT distributions across protein families. Figure 1A shows the well behaved domain family Big_1 (Pfam:PF02369) where the distribution of plDDT values is rather narrow. In Fig. 1B, we see a much broader distribution of plDDT values for the Trp_oprn_chp domain family (Pfam:PF09534). We show two exemplar structure predictions from this family in Fig. 1C to illustrate the differences that can be observed across a protein family.

Given the variability of plDDT across families we were interested to explore whether we could rescue the lower plDDT predictions using structural templates from higher plDDT models in the family. AlphaFold2 accepts protein structure templates along with evolutionary information provided as MSAs to perform structure predictions. Templates are usually derived from the PDB database (Berman *et al.* 2000) thus helping improve AlphaFold2 predictions with experimentally resolved structures, where available. Nevertheless, it has been shown that AlphaFold-derived templates can be used in combination with PDB ones, provided they have a high plDDT (Zhao *et al.* 2023). In this context, PAthreader was developed to improve AlphaFold2 template selection by looking for distant homologous proteins in both the PDB and the AFDB (Zhao *et al.* 2023).

We hypothesized that AlphaFold2 models with poor confidence predictions (plDDT lower than 70) within a protein family could be improved by using AlphaFold-derived high confidence models (plDDT higher or equal than 70) from the same family as templates. To test this, we developed a proof-of-concept workflow based on the use of ColabFold (Mirdita *et al.* 2022), which provides a versatile installation of AlphaFold. Because the plDDT values are only predictions of modelling quality, we attempt to carry out independent validations of the plDDT improvements that we observe. We have applied AF2Rank (Roney and Ovchinnikov 2022), a recently published method which implements AlphaFold2 to directly evaluate the quality of a protein model. AF2Rank consists of providing AlphaFold2 with the primary sequence of a given protein model and its structure as template after masking its side chains. A composite score is then derived which considers measures of AlphaFold2 confidence and the structural similarity between the structure predicted with this method and the input model. AF2Rank was shown to be predictive of model quality (Roney and Ovchinnikov 2022). In addition to AF2Rank, we considered the more classical MolProbity score which is widely adopted to assess protein model quality (Williams *et al.* 2018). It takes into account many variables including backbone dihedral angles, heavy atoms clashes and side chain rotamers to compose a score that can be interpreted similarly to experimental protein structure resolution, where lower values indicate higher quality.

## 2 Methods

We selected all Pfam (version 35.0) protein families that showed a clear bimodal domain plDDT distribution in the AFDB with at least 100 models (AFDB, version 4) (Mistry *et al.* 2021, Varadi *et al.* 2022). Distributions with one peak of high plDDT (average domain plDDT greater or equal to 70) and one peak of medium (average domain plDDT greater or equal to 50 and less than 70) or low (average domain plDDT less than 50) plDDT structures were selected. Fifty domain families with the most balanced distributions of low and high plDDT structures were extracted from this set (see Supplementary Fig. S1 for the plDDT distributions while the entire list of bimodal distributed domains is available in the supplementary table). For each set, a maximum of 30 proteins evenly divided between the low, medium, or high plDDT sets were chosen, prioritizing complete proteins for a total of 1460 entries. Fragment proteins, which are missing regions outside the domain of interest, were de-prioritized. Peak detection was performed on a 1–100 binned 0–1 normalized distribution of protein domain plDDTs for each domain under analysis using the function find_peaks with parameters height = 0.03 and distance = 19 from the Python module SciPy (version 1.11.2) (Virtanen *et al.* 2020). The plDDT distributions for each Pfam domain were generated using the code available at https://github.com/matthiasblum/pfam-alphafold.

Because we do not have access to the precise methodology used to predict the models in AFDB, we moved to using the ColabFold software (version 1.5.2) for our experiments so that all of our results were comparable. For each protein family, an initial structure prediction was performed with ColabFold for each family member using default parameters and relaxation without the use of templates (Mirdita *et al.* 2022). MSAs were generated with MMSeqs2 (Steinegger and Söding 2017) on the 02/2022 release of the UniRef30 database (UniProt Consortium 2018) and on the ColabFold environmental database. All confident structures (plDDT equal or greater than 70) were then selected from the pool of generated models to be used as templates for the next AlphaFold2 runs. Structure prediction with ColabFold was then run using default parameters with templates generated by the previous run using either a single sequence or the MSA as input and relaxation.

It should be noted that while we considered only the domain plDDT for the selection of the proteins from the AFDB, we later on considered the overall structure plDDT. This has been done to make sure that this remodelling methodology was not detrimental to the protein regions outside the domain of interest.

A comparison with AlphaFold3 (Abramson *et al.* 2024) was run via its dedicated web server (https://alphafoldserver.com/about).

The AF2Rank method (Roney and Ovchinnikov 2022) was run via a custom Python script to validate protein structures generated with the use of templates (https://github.com/FranceCosta/AF2Rank). To further validate protein model quality, the MolProbity score was calculated using a local implementation of the MolProbity suite, which is available on GitHub (https://github.com/rlabduke/MolProbity).

The code has been integrated in a Nextflow pipeline (version 23.04.1) (Di Tommaso *et al.* 2017) and is publicly available (https://github.com/FranceCosta/AF2Fix) along with its documentation and installation instructions. Results of the computation are available online: https://doi.org/10.5281/zenodo.13960775.

Wilcoxon signed-rank tests were performed with the wilcoxon function from the Python module SciPy (version 1.11.2). Pearson correlation was calculated using the pearsonr function from the same module.

The computations were carried out on a high performance computing cluster using NVIDIA A100 GPUs for running colabfold and AF2Rank and result in an estimated 271 kg of CO_2_ emitted for the structure recovery and validation pipeline.

**Figure 2.**
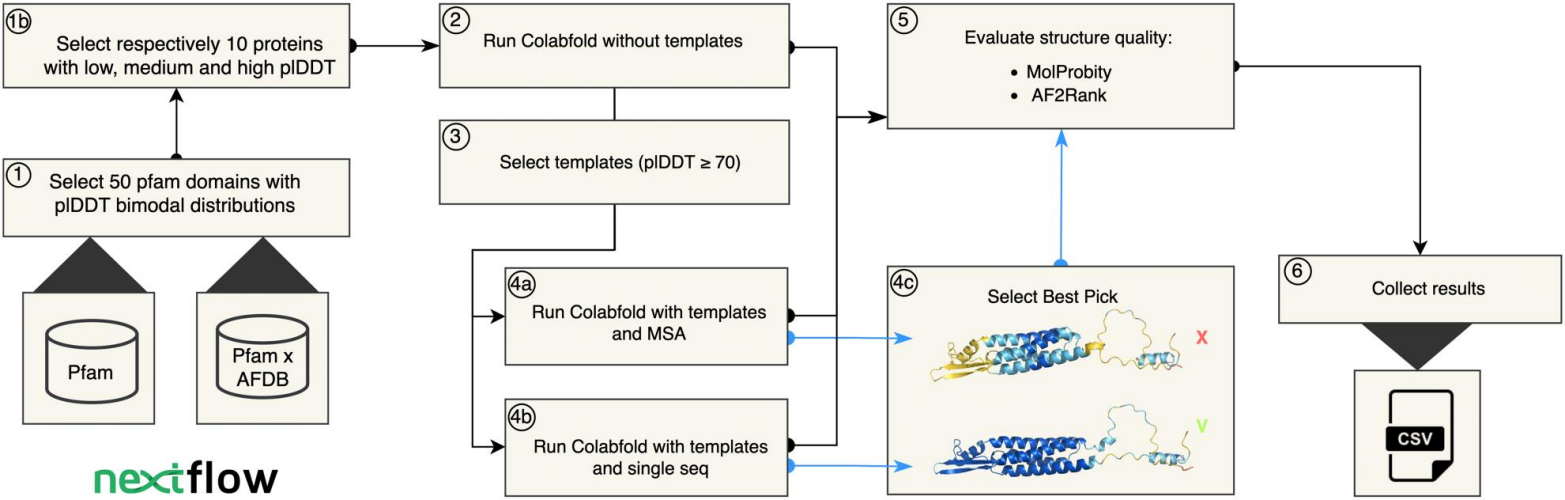
Schematic representation of the Nextflow-powered workflow (Di Tommaso *et al.* 2017) adopted in this analysis. The scheme was designed with the online draw.io app (https://www.drawio.com/).

The complete workflow can be seen in Fig. 2 and is summarized as follows:

1. 50 Pfam (Mistry *et al.* 2021) protein domains with bimodal plDDT showing one peak below the 70 confidence threshold and one above it are selected. Data are gathered from the AFDB (Varadi *et al.* 2022). (1b) 10 proteins with low (plDDT < 70), medium (70 plDDT ≤ 90), and high (plDDT ≥ 90) confidence are selected from each distribution.
2. ColabFold is run without templates on the selected proteins (Mirdita *et al.* 2022).
3. All proteins showing an average plDDT ≥ 70 are selected from the first run to be used as templates within each protein family for the subsequent runs. ColabFold is run again using the templates derived from the previous run combined with (4a) MSAs or (4b) single sequence. (4c) Best Pick proteins are selected as the proteins with the highest plDDT between runs with templates (UniProtKB: A0A7K3DYW7 shown).
4. Structure quality of the proteins before and after the adoption of templates is analysed by means of two different metrics: (i) MolProbity score (Williams *et al.* 2018), (ii) AF2Rank composite score (Roney and Ovchinnikov 2022).
5. Results are finally collected and saved in .csv format.

## 3 Results and discussion

A total of 1460 proteins from 50 different protein families were selected from the AFDB depending on their plDDT distributions, as described in the methods section. They were remodelled with ColabFold, resulting in 729 structures with low plDDT (average plDDT lower than 70). To improve these predictions, we selected all structures with high plDDT (above or equal to 70) from within each protein family to be used as templates. Templates were thus used to remodel the low plDDT predictions. In particular, they were provided to ColabFold in combination with the single sequence of the protein to remodel or with its MSA, which had been previously generated.

**Figure 3.**
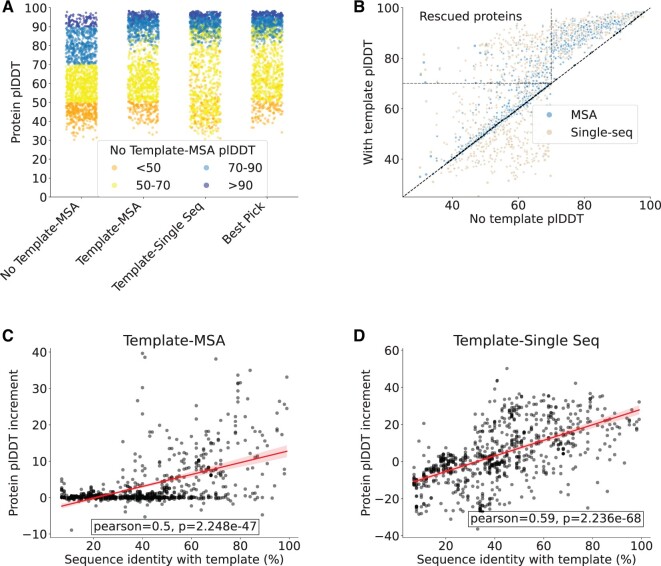
(A) Protein plDDT generated by running ColabFold with MSA and no template, with template and MSA or with template and single sequence (*N* = 1460). (B) Scatter plot showing the plDDT variation in the presence or absence of a template. Note that the “No template” condition modelling was done solely with the MSA option. Rescued proteins are those proteins for which the plDDT increased above 70 after the use of templates. As a positive control, templates were also used to remodel proteins which already exhibited a plDDT above or equal 70 without the use of templates. A plDDT improvement was expected for this set of proteins because the templates adopted coincided with the proteins to be modelled. Indeed, it has already been shown that AlphaFold2 is overly confident when it finds a perfect match between the sequence to model and the provided template (Roney and Ovchinnikov 2022). This explains why in the right hand region of the plot all the observations fall above the identity line (dotted black line). A high plDDT variability is found for proteins modelled in the presence of a template and single sequence: low plDDT values are observed when the template is too distant from the protein to be modelled. In these cases, the use of a MSA is preferable. (C) plDDT increment in proteins modelled with a template and MSA or with templates and single sequence (D) correlates with the sequence identity with the closest template used (*N* = 729). 95% confidence interval is shown in the trend lines. Sequence identity was calculated with PairwiseAligner from the Biopython module using BLOSUM62 as substitution matrix and global alignment option.

We observed a statistically significant improvement (Wilcoxon paired, one-sided; *P* < 0.001) of the average plDDT for the set of 729 (out of 1460 proteins) low confidence protein structures in either single sequence and MSA modes. In particular, the use of templates coupled with a single sequence input led to an increase of the plDDT on average coupled to a marked variability (4.2 ± 15.5). In particular, it provoked a strong decrease of the plDDT for a subset of proteins, as illustrated in Fig. 3A and B. The adoption of templates in combination with the MSA induced a more modest improvement of the plDDT (3.5 ± 7.1) with most of the proteins exhibiting only a marginal increase in plDDT while only very few proteins had a decrease in plDDT. This data suggest that templates have a stronger influence on the plDDT when combined with a single sequence input (Wilcoxon paired, one-sided; *P* < 0.001). When a MSA is used instead, the templates contribute less or are even ignored completely in some cases. Based on our findings, we introduced an additional category called “best pick.” This category comprises the same proteins found in the previously mentioned classes. However, for each protein, we selected the structural model that achieved the highest plDDT score, regardless of whether it came from the template-MSA or the template-single sequence approach. “Best pick” benefits from structures which increase plDDT by the use of templates in the single sequence mode and removes the structure predictions that decreased in plDDT score replacing them with the MSA-based models. When considering the “best pick” models, the average plDDT increase is higher than the previous classes (8.7 ± 10.9). The relationship between plDDT before and after the introduction of templates can be observed in Fig. 3B. This plot highlights how for some proteins the use of templates with a single sequence can be deleterious, further supporting the introduction of “best pick” proteins. The dotted region of the graph in the top left demarcates the “rescued proteins.” These proteins showed an improvement of their plDDT above the confidence threshold of 70 when templates were used. Two hundred and forty-five out of 729 entries fall into this category.

We additionally investigated whether the improvement in plDDT occurs in disordered regions (Supplementary Figs S4 and S5), finding that it mostly interests regions predicted to be ordered by IUpred2A (Mészáros *et al.* 2018).

The sequence identity with the template plays an important role in determining the model improvement, with the highest plDDT increase being associated with high protein-template sequence similarity both when using a MSA (Fig. 3C) or a single sequence (Fig. 3D). In particular, when the sequence similarity with the template falls below 30% sequence identity, either there is no plDDT improvement when a MSA is used or the confidence decreases when a single sequence is adopted. Moreover, the identity with the template is a stronger driver of confidence increment for the single sequence mode (*r* = 0.59, *P* < 0.001) compared to the MSA mode (*r* = 0.5, *P* < 0.001).

When comparing ColabFold results with those from the AlphaFold database, we found a good agreement in the plDDTs derived from the two methods (*r* = 0.63, *P* < 0.001). In addition to this, we observed a significant correlation between MSA depth and plDDT when no templates are used (*r* = 0.12, *P* < 0.001), indicating that MSA size is a possible cause for AlphaFold2 generating models with low plDDT for some family members in the first place (Supplementary Fig. S2).

We investigated whether AlphaFold3 alone was capable of improving the plDDT by running it on a subset of 20 randomly selected rescued proteins finding no statistically significant plDDT improvement compared to our baseline (Supplementary Fig. S6). In addition to this, we tested the use of multiple ColabFold runs with different seeds producing a total of 50 different models per protein on a subset of 50 randomly selected rescued proteins. No statistically significant improvement was detected as well (Supplementary Fig. S7).

**Figure 4.**
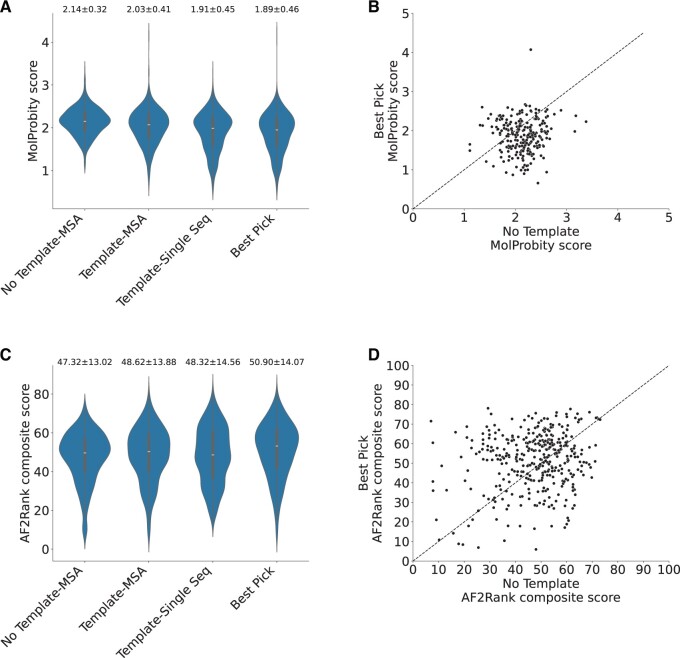
(A) Violin plot showing the distribution of MolProbity scores in different conditions (*N* = 728). (B) Scatter plot showing the distribution of MolProbity scores in the conditions “No Template” and “Best Pick.” Best Pick models have higher quality as shown by the lower MolProbity scores compared to No Template ones and confirmed by statistical analysis (Wilcoxon paired, one-sided; *P*-value: 2.61e−45). The dotted black line represents the identity line. (C) Violin plot showing the distribution of AF2Rank composite scores in different conditions (*N* = 728). (B) Scatter plot showing the distribution of AF2Rank composite scores in the conditions “No Template” and “Best Pick.” In this case, the higher quality Best Pick models is less evident from the distribution but it is nonetheless statistically significant (Wilcoxon paired, one-sided; *P*-value: 5.18e−19). The dotted black line represents the identity line. Average value and standard deviation are reported on top for (A) and (C).

In order to assess the protein structure quality after remodelling with templates, we adopted two different methods: (i) score using AF2Rank, (ii) Molprobity score. Both measures demonstrated that Best Pick models correspond to high-quality structures (Wilcoxon paired, one-sided; *P* < 0.001), as illustrated in Fig. 4. These two validation studies showed that structures associated with an increase in AlphaFold2 confidence are effectively subject to an improvement in model quality. These methods cannot provide an absolutely unbiased way to determine structure quality, for which experimentally derived models would represent the best option. These metrics can nonetheless serve as an indication of model improvement. Therefore, the plDDT is a good metric of protein quality in this context and can thus be adopted to identify better models.

We are interested in improving the efficiency of our computations and so we investigated the running time of ColabFold with and without template structure. We found that the use of templates does lead to faster calculation of the structural models. We found that using both template and MSA took 37.8% less time than just using an MSA, whereas, using templates without MSA took 60.6% less time (Supplementary Fig. S3A). Therefore, rather surprisingly running the Best Pick option of running ColabFold with both the MSA switched on and off took almost the same amount of time as running the predictions without templates. These results can be explained by our observation that structure models also converge more rapidly when templates are used (Supplementary Fig. S3B).

We have shown that it is effectively possible to improve low confidence models using AlphaFold2 generated templates from the same family and that increased plDDT can be used to infer structure model improvement. Overall, the increase of the plDDT is dependent on the similarity with the templates adopted, suggesting that more refinement in the selection of the templates might further improve the quality of the rescued protein models. Nevertheless, the reason why some proteins are predicted with low plDDT in the first place remains uncertain. We have found only a very weak correlation between MSA depth and plDDT which suggests that other factors, such as the quality of the alignments, may be playing a role. The poor performance of AlphaFold in the first place is therefore likely due to the low quality and depth of the MSAs, which can widely differ for different proteins with only one domain in common.

Finally, it is worth noting that our analysis was biased towards ordered protein domains. First, we used Pfam families as input which are strongly biased against disordered regions of proteins (Mistry *et al.* 2021). Second, by excluding those families which did not show any high confidence candidates we likely avoid disordered proteins. Disordered proteins are unlikely to benefit from template-based remodelling.

## 4 Conclusions

Protein models generated by AlphaFold2 exhibit a heterogeneity of confidence as measured with the plDDT metrics. This is especially true within some protein domain families. Here, we have shown that a portion of these proteins can be effectively rescued by coupling protein modelling with high plDDT templates obtained from within the same protein family. In particular, selecting the proteins with the highest plDDT out of the single sequence mode and the MSA mode yielded the best results in terms of validation metrics. We were surprised to find that some proteins only exhibited a strong increase in plDDT when the template was adopted in combination with a single sequence, indicating that the use of the MSA might mask the structural information provided by a template.

The main cause of AlphaFold failure is likely the low quality and low depth of MSAs in the first place. For this reason, the use of templates may be especially valuable when using AlphaFold2 on proteins from organisms with little representation in the sequence databases, or perhaps rapidly evolving taxa such as bacteriophage. The recently created Big Fantastic Virus Database (BFVD) (Kim *et al*. 2024) could be particularly useful in this context to provide templates for phage structure prediction.

We also note that using templates where available is suitable for large scale modelling as their adoption can improve computation efficiency. This in turn could reduce the carbon footprint of the calculations.

## Supplementary Material

vbae188_Supplementary_Data

## Data Availability

The data underlying this article are available in *Zenodo* at https://doi.org/10.5281/zenodo.13960775.
