# Supplementary material for: Keeping it in the family: using protein family templates to rescue low confidence AlphaFold2 models
Source: Bioinform Adv. 2024 Nov 25;4(1):vbae188. doi: 10.1093/bioadv/vbae188 (PMC11630841; doi:10.1093/bioadv/vbae188)
Supplement: vbae188_Supplementary_Data [file vbae188_supplementary_data.pdf]

1 Supplementary figures

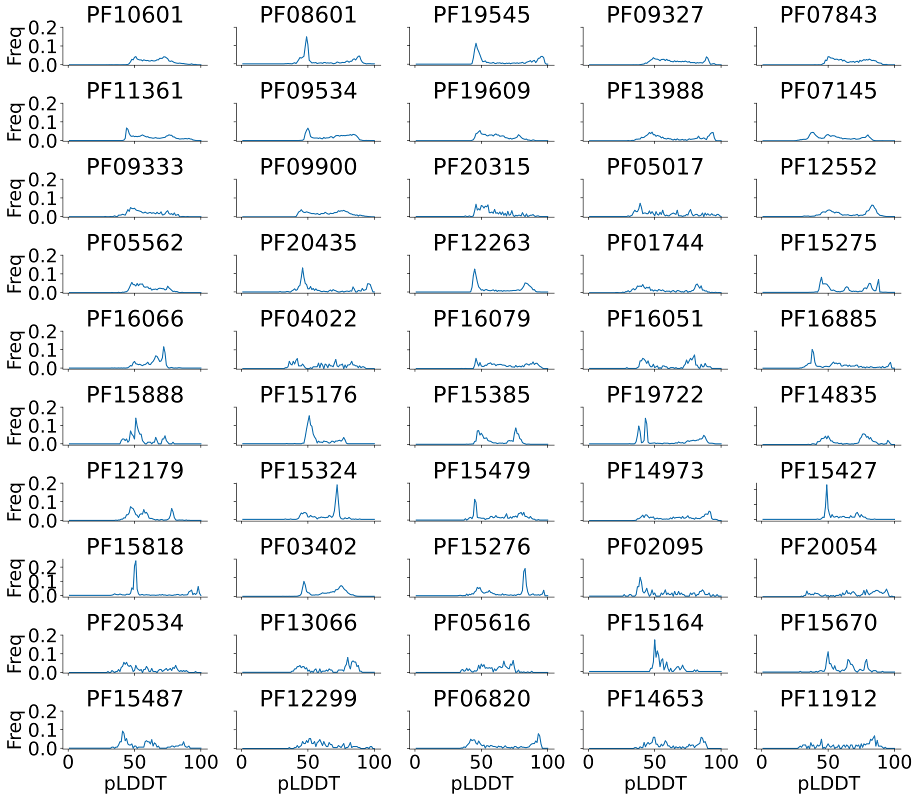

Figure S1: plDDT frequency distributions of the 50 Pfam domains considered in this study. Data obtained from the AFDB (Varadi et al., 2022, 2024). The entire set of bimodal distributions can be seen in the supplementary table 1.

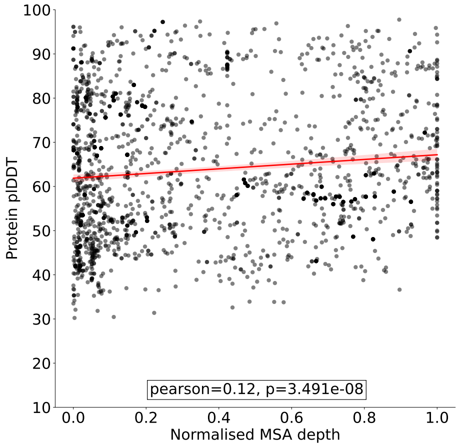

Figure S2: Correlation between plDDT and the normalised number of sequences per MSA. 95% confidence interval is shown in the trend line. The length of the deepest MSA within each domain among the selected proteins was used as a normalisation factor.

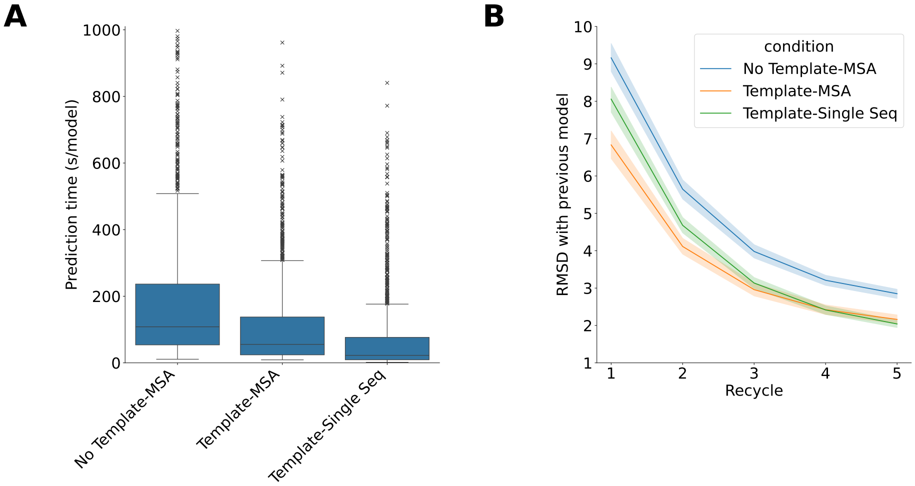

Figure S3: (A) Execution time of ColabFold structure prediction when modelling without the template or with the template and MSA or template and single sequence. Each value is the average running time calculated across 5 different predictions (N=1460). (B) ColabFold convergence when using no template or a template and either single sequence or MSA mode. The Root Mean Square Deviation (RMSD) with the previous model at every recycle is shown. Confidence intervals are displayed.

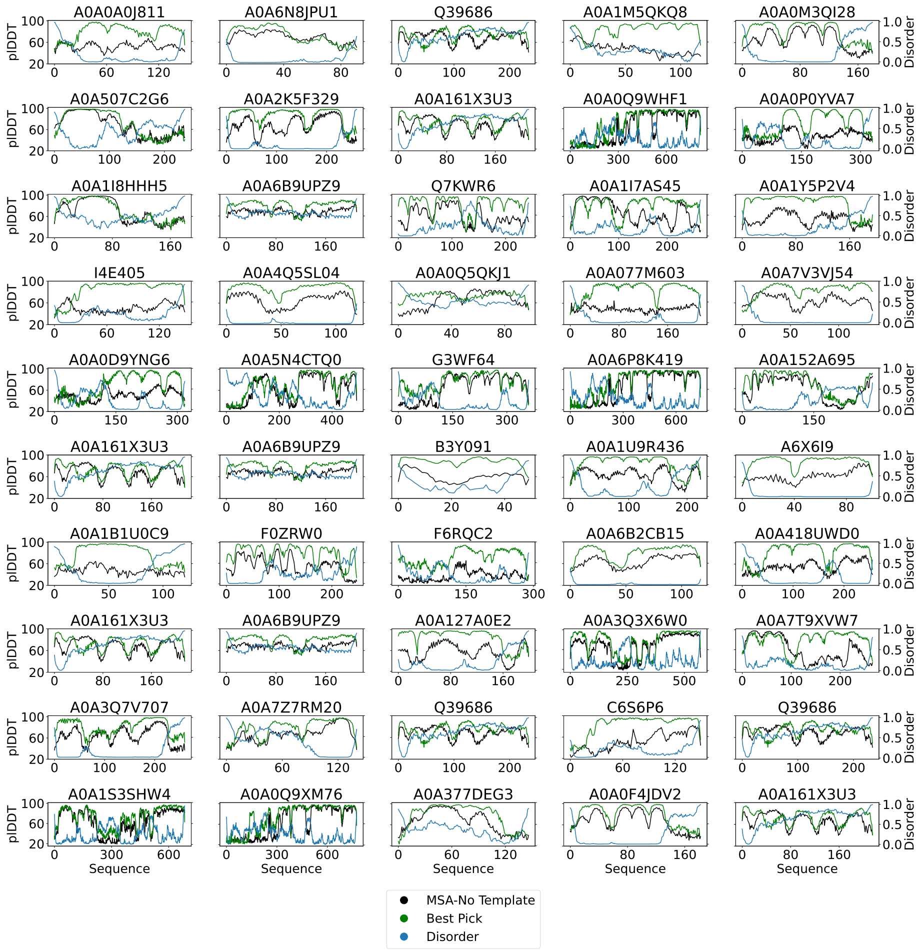

Figure S4: plDDT before rescue (MSA-No Template) and after rescue (Best Pick) of a group of 50 randomly selected rescued proteins. Disorder probability as measured by IUpred2A is shown as well. Disorder regions are not, or they are only marginally, subject to plDDT increase.

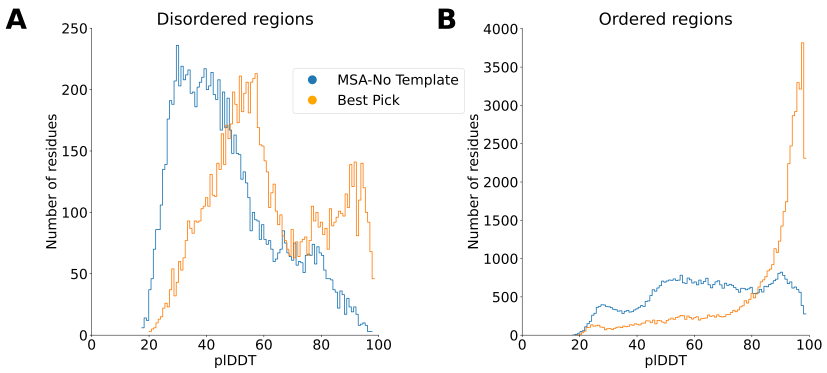

Figure S5: plDDT distributions before and after rescue in disordered (A) and ordered (B) regions as measured by IUpred2A, where disorder is assigned for probabilities above 0.5. While disordered regions are subject to a plDDT increment, they represent a minimal fraction of rescued proteins. The increment in plDDT is prevalent for ordered regions.

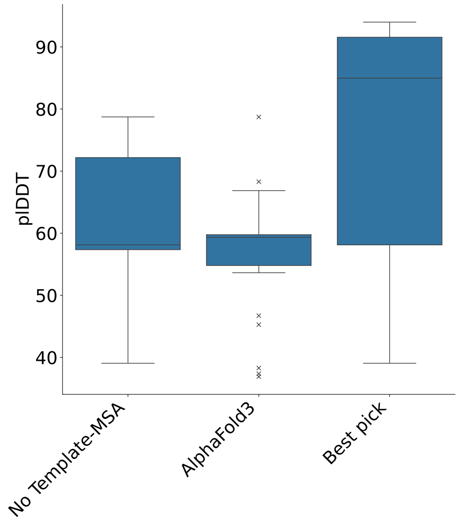

Figure S6: ColabFold (No Template-MSA), AlphaFold3 and ColabFold with custom templates (Best Pick) - plDDT values calculated on 20 proteins with initial low plDDT (<70 as measured on the AFDB models) randomly selected from our dataset. AlphaFold3 models showed similar plDDT values to ColabFold ones without the use of templates (Wilcoxon paired, two-sided; p=0.43). Best pick models on the other hand, show a statistically significant higher plDDT compared to both ColabFold models with no template (Wilcoxon paired, two-sided; p¡0.001) and AlphaFold3 models (Wilcoxon paired, two-sided; p<0.001). Corrected p-value = 0.016.

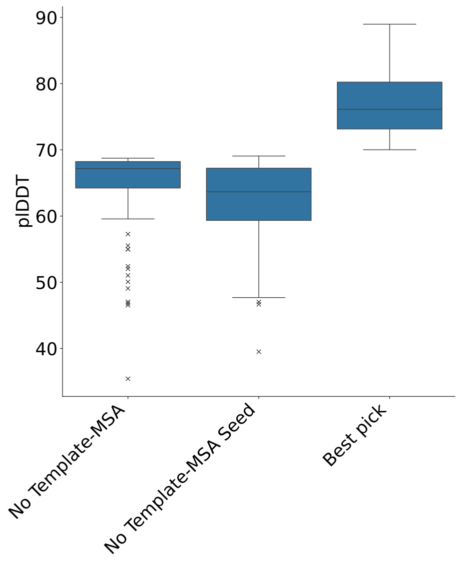

Figure S7: plDDT comparison between best pick models (best model between MSA and single sequence, using templates) and models generated without templates and MSA or with the same setting but by re-running the prediction 10 times with a different seed each time (and 5 models per seed) and selecting the best model among each pool of 50 models per protein. Seeds ranged from 0 to 9. The comparison was run on 50 different proteins randomly selected among rescued models. There is no statistically significant difference between No Template-MSA and No Template-MSA Seed (Wilcoxon paired, two-sided; $p=0.32$) while the difference between No Template-MSA/No Template-MSA and Best pick are both statistically significant (Wilcoxon paired, two-sided; $p<0.001$). Corrected p-value = 0.016.

References

M. Varadi, S. Anyango, M. Deshpande, S. Nair, C. Natassia, G. Yordanova, D. Yuan, O. Stroe, G. Wood, A. Laydon, A. Žídek, T. Green, K. Tunyasuvunakool, S. Petersen, J. Jumper, E. Clancy, R. Green, A. Vora, M. Lutfi, M. Figurnov, A. Cowie, N. Hobbs, P. Kohli, G. Kleywegt, E. Birney, D. Hassabis, and S. Velankar. AlphaFold Protein Structure Database: massively expanding the structural coverage of protein-sequence space with high-accuracy models. *Nucleic Acids Research*, 50(D1):D439–D444, Jan. 2022. ISSN 0305-1048, 1362-4962. doi: 10.1093/nar/gkab1061. URL `https://academic.oup.com/nar/article/50/D1/D439/6430488`.

M. Varadi, D. Bertoni, P. Magana, U. Paramval, I. Pidruchna, M. Radhakrishnan, M. Tsenkov, S. Nair, M. Mirdita, J. Yeo, O. Kovalevskiy, K. Tunyasuvunakool, A. Laydon, A. Žídek, H. Tomlinson, D. Hariharan, J. Abrahamson, T. Green, J. Jumper, E. Birney, M. Steinegger, D. Hassabis, and S. Velankar. AlphaFold Protein Structure Database in 2024: providing structure coverage for over 214 million protein sequences. *Nucleic Acids Research*, 52(D1):D368–D375, Jan. 2024. ISSN 0305-1048, 1362-4962. doi: 10.1093/nar/gkad1011. URL `https://academic.oup.com/nar/article/52/D1/D368/7337620`.
